# Claustrum sparing sign in seronegative limbic encephalitis

**DOI:** 10.1016/j.ensci.2023.100465

**Published:** 2023-05-16

**Authors:** Abeer Sabry Safan, Mohammad Al-Termanini, Mohamed Abdelhady, Yasir Osman, Abdel-Nasser Y. Awad Elzouki, Ahmed Lutfe Abdussalam

**Affiliations:** a Department of Neurology, Neurosciences Institute, Hamad Medical Corporation, Doha, Qatar; b Department of Neuroradiology, Neurosciences Institute, Hamad Medical Corporation, Doha, Qatar; c Department of Internal Medicine, Hamad Medical Corporation, Doha, Qatar; d Department of Medical Intensive Care Unit, Hamad Medical Corporation, Doha, Qatar

**Keywords:** Limbic encephalitis, Seizure, Claustrum sparing sign, Seronegative

## Abstract

**Background:**

Limbic encephalitis (LE) is a rare variant of autoimmune encephalitis. It often manifests with subacute neuropsychiatric symptoms of agitation, delusions, variable seizure semiology, and short-term memory loss. Seronegative limbic encephalitis can pose a diagnostic conundrum owing to its inadequately understood pathophysiology.

**Case presentation:**

We report a rare case of a young male with subacute neuropsychiatric manifestations of delusions, agitations and seizures. He was diagnosed with seronegative limbic encephalitis (SNLE). Brain MRI demonstrated bilateral Claustrum sparing sign. An EEG showed continuous left-sided epileptiform discharges in periodic to predominantly left middle temporal. Patient condition gradually improved with pulsed methylprednisolone, intravenous immunoglobulins and anti-seizure medications.

**Conclusion:**

Claustrum remains one of the least understood neuroanatomical structures. Claustrum sign has been reported in febrile infection-related epilepsy syndrome (FIRES), LE, and autoimmune refractory epilepsy. To the best of our knowledge, we report the first case in literature with Claustrum sparing sign in seronegative Limbic Encephalitis. Further experimental models and researches are warranted to better understand the unique function of the claustrum and unravel possible other attributable auto-antibodies, which could alter treatment and prognosis.

## 1 Background

Limbic encephalitis was first described in 1968 by Brievely and Corsellis with predominate limbic system involvement, particularly the hippocampus and amygdala. [1] Cardinal manifestation of limbic encephalitis (LE) can vary on a spectrum from amnesia, behavioral changes, seizures, and altered level of consciousness, with some cases progress to fatal none refractory status epilepticus and coma. [1,2]

Hallmark radiological findings of Autoimmune limbic encephalitis (ALE) is involvement of the mesial temporal lobes and insula which is bilateral in 60% of the cases [ 4]. Atypical radiological presentation of seronegative limbic encephalitis impose a clinical challenge and could delay vital therapy [4,7]. Herein we report a 30-years old male who presented with paroxysmal abdominal pain with subsequent neuropsychiatric manifestation diagnosed as seronegative limbic encephalitis with atypical bilateral extreme external capsule signal intensity, hence giving the claustrum sparing appearance.

## 2 Case presentation

A 30-year-old right-handed gentleman with unremarkable past medical and family history presented to the Emergency Department (ED) with nine days history of fever associated with headache, neck stiffness, and blurring of vision. Headache described as holocephalic, non-radiating pressure like with photophobia and phonophobia alongside paroxysmal sharp abdominal pain, stabbing in nature, that lasts for few seconds to one minute with nausea and vomiting. Upon admission, he developed two episodes of generalized tonic-clonic seizures (GTCS) lasting one minute each aborted with 2 mg (mg) of intravenous (IV) lorazepam and IV 2-g loading dose of levetiracetam, following by 1000 mg twice daily.

His vital signs upon arrival to Hamad General Hospital ED revealed a temperature of 38 °C, respiratory rate of 17 breaths per minute, blood pressure of 129/62 mmHg (with no postural variation), and oxygen saturation of 98% on room air. He appeared agitated with persecutory delusions, preservation, and echolalia. Mini-mental state examination (MMSE) score was 15/30 with impaired orientation, registration, attention and recall. Neurological examination was none focal with negative Kernig's sign and normal optic disc exam. No observed extrapyramidal signs or focal neurological deficits aside from biceps and patellar hyperreflexia with clonus (4/4) with bilateral extensor plantar responses.

Initial work up revealed unremarkable computed tomography (CT) head. Lumbar puncture (LP) showed grossly clear cerebrospinal fluid (CSF) with lymphocytic pleocytosis of 97% (Normal range: 40–80%) glucose 3.55 mmol/L (Normal range: 2.22–3.89), protein 0.32 g/L (Normal range: 0.15–0.45). Acyclovir and ceftriaxone are initiated empirically for a preliminary diagnosis of meningio-encephalitis. SARS-CoV-2 (COIVD-19) nasopharyngeal swab PCR was negative. Day two of admission, he developed a flurry of GTCS that progressed into status epilepticus, warranting midazolam infusion and admission to the Medical Intensive Care Unit (MICU). Anti-seizure medication were optimized and seizure control attained with levetiracetam 1 g BID, Lacosamide 200 mg BID, and Clobazam 10 mg BID and midazolam infusion was stopped after 24-h with regain of consciousness.

**Fig. 1:**
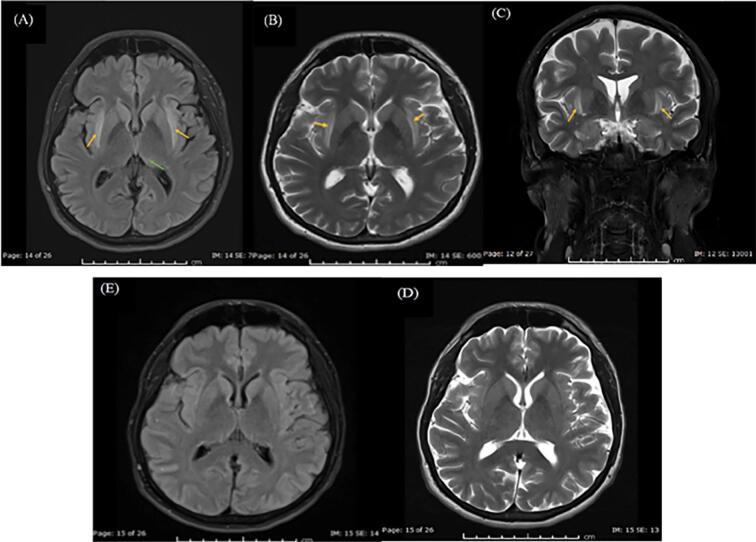
MRI head. Axial Fluid Attenuation Inversion Recovery (FLAIR), [B-C] Axial and Coronal T2-weighted images: Bilateral symmetric external and extreme capsules high T2-signal intensity with sparing of the intervening claustrum (orange arrows) bilaterally and subtle focus of high signal intensity left thalamus (Green arrow). 18 days follow up MRI [D] FLAIR images [E] T-2 weighted images: Significant regression with near total resolution of previously seen bilateral symmetric external and extreme capsules high T2-signal intensity as well as the subtle focus of high signal intensity at left thalamus.

Initial magnetic resonance imaging (MRI) brain, demonstrated bilateral symmetric external and extreme capsules, left thalamus high T2 signal / fluid attenuated inversion recovery (FLAIR) with sparing of bilateral claustrum (Fig. 1 A-C). There was no significant abnormal post-contrast enhancement or other noted anomality. Possible differential diagnosis proposed was autoimmune limbic encephalitis, acute intermittent porphyria owing to the recurrent paroxysmal abdominal pain with co-current neuropsychiatric manifestations.

An electroencephalogram (EEG) showed continuous left-sided epileptiform discharges in the form of periodic to predominantly left middle temporal Area and lesser extend anterior temporal area T reminiscent of Left Temporal epileptiform focus, suggestive of left limbic encephalitis [12]. Autoimmune encephalitis serum and CSF panel were sent to Mayo clinic laboratory which was negative and malignancy screening showed unremarkable CT chest, abdomen and pelvis (PAN-CT) reaching to a working diagnosis of seronegative limbic encephalitis (SNLE).

Management encompassed a multidisciplinary approach with pulsed IV methylprednisolone (1 g/day for five days) followed by IV immunoglobulin (IVIG) 2 g/kg over five days. Upon completing his immunotherapy, personality changes, abdominal pain, and seizures have almost resolved with residual impaired short-term memory with MMSE 27/30 (impaired recall). Follow up EEG obtained on day fourteen of admission showed left posterior temporal epileptiform discharges on top of mild encephalopathy. A 3-week Follow-up MR displayed significant regression (almost total resolution) of previously noted symmetric signal abnormality at bilateral external and extreme capsules as well as at the left thalamus, as shown in Fig. 1 D-E. Subsequent follow ups in neurology clinics showed well controlled seizures with resolution of neuropsychiatric manifestations and planned to gradually taper off his anti-seizure medications (Levetiracetam 1 g BID, Lacosamide 200 mg BID, and Clobazam 10 mg BID).

## 3 Discussion and conclusion

Pathophysiology of limbic encephalitis is not fully discernable and it can be subclassified according to its underlying etiology as paraneoplastic or none-paraneoplastic limbic encephalitis (NPLE) [5]. Commonly described tumors with paraneoplastic limbic encephalitis, include small cell lung cancer (SCLC), Anti-Ta antibody in germ-cell testicular tumors, breast cancer, Hodgkin's lymphoma, immature teratoma, and thymoma. [[1], [2], [3]] Another approach of classifying limbic encephalitis is related to antibody target, whether extracellular or intracellular antigens, with the latter, more frequently associated with a paraneoplastic origin. [7] For instance, Anti- PNMA2 (formerly anti-Ma2/Ta) antibodies target intracellular antigens associated with PNLE in testicular tumors, while Anti-GAD (Glutamic acid decarboxylase) antibodies are presenting with classic limbic involvement and stiff person syndrome, without usual evidence of tumors [ 7,8].

Nonetheless, it is not unusual for LE to have a none-paraneoplastic or seronegative variant relative to the autoimmune encephalitis mosaic six-screen known to date which raises the possibility of undiscovered auto-antibodies as the culprit. [6,7] Seizures' semiology in acute limbic encephalitis (ALE) vary on a spectrum from focal with variable alteration in awareness that could progress to secondary generalization due to the medial temporal lobe involvement making it resistive to a wide array of anti-seizure drugs (ASD). [4,5] The diagnostic criteria of limbic encephalitis; includes subacute neuropsychiatric manifestation, CSF findings suggestive of inflammation, typical MRI, and EEG abnormality, and exclusion of alternative causes [5]. In 2011, treatment options reconnoitered in Eric Lancaster laboratory for paraneoplastic and autoimmune LE with emphasis on first line treatment to encompass pulsed steroids, intravenous immunoglobulin (IVIG) and plasma exchange [7]. Refractory cases warrant use of immunosuppression with use of rituximab and cyclophosphamide as second-line options. [7] Nonetheless, there was not enough compelling evidence on seronegative variants treatment algorithm [7]. Our patient was treated successfully with similar protocol for autoimmune LE with IV methylprednisolone and IVIG, with resolution of presenting symptoms and radiological signs on interval follow ups.

Radiologically speaking, the MRI head can be normal or show mild abnormality in LE causing a clinical-radiological paradox, as some radiological abnormality might not correlate with the clinical presentation [8]. However commonly observed classical radiological features encompass a high T2/FLAIR signal intensity in the mesial temporal lobes, basal ganglia, and limbic system which is symmetrical in 60% of the cases, although often observed findings are asymmetric involvement. [7,8] In contrast to mimickers of LE, lateral temporal lobes, basal ganglia, and insula are less commonly affected compared to what is observed in herpes simplex virus (HSV) encephalitis [9]. Thalamic involvement is considered a rare finding in LE with most reported cases in the literature were associated with anti-*N*-methyl-d-aspartate receptor (NMDAR) encephalitis [9,13]. Interestingly our patient had asymmetrical MRI findings with left thalamic high T2 signal sparing temporal lobes and the limbic system.

The claustrum is one of the multi-modal processing structure that is housed between the cortex and the putamen separated by the extreme and external capsule. [11] To date, this anatomical structure despite constituting the specialized network of microcircuits; its pathophysiological impact in limbic encephalitis is not fully comprehended. [11,12] Bilateral claustra T2/FLAIR hyperintensity, the so-called Claustrum sign, is frequently observed acute phase in conditions attributed with refractory seizures; as observed in febrile infection-related epilepsy syndrome (FIRES) and para-infectious encephalitis in COVID-19. [11] In chronic phases if complete recovery in such seizure disorders was not achieved claustra atrophy is observed with corresponding hypointensity [11,12].

Claustrum hyperintensity has been described in number of autoimmune encephalitis and epilepsy disorders [11]. However, claustrum sparing sign has never been reported in seronegative limbic encephalitis. We presume this is the first case in literature to describe such sign as it was not described earlier with any other pathology [10]. Neurologist and radiologist must be aware of such rare sign, as it can be clinically challenging. We believe such radiological sign would warrant further research to better understand such anatomical dilemma and its clinical implications.

## Ethics approval and consent to participate

This case report was approved by the Hamad Medical Corporation's Medical Research Center (Protocol number: MRC-04-21-456).

## Consent for publication

Written informed consent was obtained from the patient for publication for this case report and any accompanying images. A copy of the written consent is available for review by the Editor of this journal.

## Funding

Qatar National Library funded the open access publication fees of this case report.

## Author contributions

Writing the initial draft of the manuscript: AS, MT, MA.

Conceptualization and supervision: AE, YO.

Medical management of the case: AS, MT, YO, AL.

Revising the manuscript critically and literature review: AS< MT, MA, MA, AE, YO.

The first authors (AS and MT) contributed equally to the writing and preparation of this article. AS, MT, and MA have written the initial draft of the manuscript and performed the literature review. The draft was revised and updated by AS, MT, MA with supervision from AZ and YO. AS, MT, YO, ALA were part of the medical treating team. All the authors critically reviewed the initial and the final draft of the manuscript and approved it for submission.

## Credit author statement

**Table:** 

Name	Location	Contribution
Abeer Sabry Safan	Neurosciences Institute, Hamad Medical Corporation, Doha, Qatar	Writing the initial draft of the manuscript, medical management of the case, revising the manuscript critically and literature review.
Mohammad Al-Termanini	Internal Medicine, Hamad Medical Corporation, Doha, Qatar	Writing the initial draft of the manuscript, medical management of the case, revising the manuscript critically and literature review.
Mohamed Abdelhady	Neuroradiology, Neurosciences Institute, Hamad Medical Corporation, Doha, Qatar	Obtaining the radiological images and labels, with revising the manuscript critically and literature review.
**Abdel-Nasser Y. Awad Elzouki**	Internal Medicine, Hamad Medical Corporation, Doha, Qatar	Conceptualization and supervision, medical management, revising the manuscript critically and literature review.
**Yasir Osman**	Neurosciences Institute, Hamad Medical Corporation, Doha, Qatar	Conceptualization and supervision, medical management, revising the manuscript critically and literature review.
**Ahmed Lutfe Abdussalam**	Medical Intensive care unit, Hamad Medical Corporation, Doha, Qatar	supervision, medical management.

## CRediT authorship contribution statement

**Abeer Sabry Safan:** Conceptualization, Writing - original draft. **Mohammad Al-Termanini:** Conceptualization, Writing - original draft. **Mohamed Abdelhady:** Conceptualization, Writing - review & editing. **Yasir Osman:** Conceptualization, Writing - review & editing. **Abdel-Nasser Y. Awad Elzouki:** Conceptualization, Writing - review & editing. **Ahmed Lutfe Abdussalam:** Writing - review & editing.

## Declaration of Competing Interest

The authors have no conflicts of interest to disclose.

## Data Availability

The datasets used and/or analyzed during the current study are available from the corresponding author on request.
